# On Filing Teeth for Superficial Decay

**Published:** 1860-01

**Authors:** Jno. McCalla


### ARTICLE V

### *On Filing Teeth for Superficial Decay.*

By Jno. McCalla, D. D. S.

In looking over the proceedings of the late American Dental Convention at Niagara, we find the following question proposed for consideration: "under what circumstances, and in what manner, should teeth be filed to remove superficial decay, or preparatory to filling, to leave them the most secure from future decay?" The proposition assumes that teeth *should* be filed for superficial decay, but the manner in which it should be performed, and the circumstances calling for the operation, was left for the convention to determine. The question was not kept clearly in view, in the discussson which followed, and the subject was finally disposed of by a vote in favor of filing for the removal of superficial decay on the approximal surfaces of teeth. From this decision, we beg leave to dissent, in so far as it is intended to furnish a *rule* for those who look to our dental conventions for sound doctrine in regard to dental practice. In deciding when to file for superficial decay, we should be governed by the following considerations: first, the age and temperament of the individual; second, the habits in regard to cleanliness of the mouth, and third, the extent of decay to be removed. In regard to the first, or physical characteristics, we shall adopt the classification of "Harris" as the standard, and remark, that where the conditions are favorable, teeth of the first class are fit subjects for the file, when judiciously employed; by judicious, we wish to be understood, that the decay should not extend through the enamel, that the least possible space should be made externally consistent with the thorough removal of all the decay, the sharp edges of the aperture removed, and the parts left, not only smooth, but completely condensed and burnished. We met with a case some years ago, answering in a degree to this description, which had been filed for superficial decay, by Dr. Leonard Koecker, thirty-eight years before; the teeth were somewhat mutilated, but in a perfect state of preservation, such cases however may be considered exceptions to the rule; in the same space of time that we have met with a few such instances, hundreds have come to our knowledge of irremediable injury having been produced by similar operations. In teeth of a less degree of density, the liability to farther decay is increased in proportion to the degree, while the space left for the formation of a suitable cavity for filling is so much abridged by the previous filing, as to bring us into dangerous, if not fatal, proximity to the pulp, an event not beyond remedy it is true, yet always to be deplored.

It has been adduced in favor of filing for superficial decay, that the Brahmins of India file their teeth even at an early age, yet they seldom, or never decay. When we take into consideration that the care which they bestow upon their teeth is inculcated from infancy with the binding obligation of a religious duty, the argument loses much of its force; few of our patients, with all the advantages of a higher civilization, could be induced to devote an hour or more every morning, as they do, to the purification of the mouth. It is well known too that the teeth of Americans rank much lower in the physical scale, than those of the people just alluded to; with the causes producing this difference we have nothing to do at present, but offer the fact as another argumet in favor of our position. The result of our observations in relation to the operation under consideration is, that while we look upon the file as indispensable in dental practice, there are comparatively few cases of superficial decay presented to our notice calling for its employment, we prefer to wedge teeth apart, and if they are not sufficiently decayed for filling, cleanse and burnish the parts, enjoining strict cleanliness and the importance of having them frequently inspected.

